# From static to temporal network theory: Applications to functional brain connectivity

**DOI:** 10.1162/NETN_a_00011

**Published:** 2017-06-01

**Authors:** William Hedley Thompson, Per Brantefors, Peter Fransson

**Affiliations:** Department of Clinical Neuroscience, Karolinska Institutet, Stockholm, Sweden

**Keywords:** Resting-state, Temporal network theory, Temporal networks, Functional connectome, Dynamic functional connectivity

## Abstract

Network neuroscience has become an established paradigm to tackle questions related to the functional and structural connectome of the brain. Recently, interest has been growing in examining the temporal dynamics of the brain’s network activity. Although different approaches to capturing fluctuations in brain connectivity have been proposed, there have been few attempts to quantify these fluctuations using temporal network theory. This theory is an extension of network theory that has been successfully applied to the modeling of dynamic processes in economics, social sciences, and engineering article but it has not been adopted to a great extent within network neuroscience. The objective of this article is twofold: (i) to present a detailed description of the central tenets of temporal network theory and describe its measures, and; (ii) to apply these measures to a resting-state fMRI dataset to illustrate their utility. Furthermore, we discuss the interpretation of temporal network theory in the context of the dynamic functional brain connectome. All the temporal network measures and plotting functions described in this article are freely available as the Python package Teneto.

## Author Summary

Temporal network theory is a subfield of network theory that has had limited application to date within network neuroscience. The aims of this work are to introduce temporal network theory, define the metrics relevant to the context of network neuroscience, and illustrate their potential by analyzing a resting-state fMRI dataset. We found both between-subjects and between-task differences that illustrate the potential for these tools to be applied in a wider context. Our tools for analyzing temporal networks have been released in a Python package called Teneto.

It is well known that the brain’s large-scale activity is organized into networks. The underlying organization of the brain’s infrastructure into networks, at different spatial levels, has been dubbed the brain’s *functional and structural connectome* (Sporns, 2009; Sporns, Tononi, & Kotter, 2005). Functional connectivity, derived by correlating the brain’s activity over a period of time, has been successfully applied in both functional magnetic resonance imaging (fMRI; Greicius, Krasnow, Reiss, & Menon, 2003; Fransson, 2005; Fox et al., 2005; Smith et al., 2009) and magnetoencephalography (MEG; de Pasquale et al., 2010; Brookes et al., 2011; Hipp, Hawellek, Corbetta, Siegel, & Engel, 2012), yielding knowledge about functional network properties (Buckner et al., 2009; Power et al., 2011; Power, Schlaggar, Lessov-Schlaggar, & Petersen, 2013; Nijhuis, van Cappellen van Walsum, & Norris, 2013) that has been applied to clinical populations (Fox & Greicius, 2010; Zhang & Raichle, 2010).

In parallel to research on the brain’s connectome, there has been a focus on studying the dynamics of brain activity. When the brain is modeled as a dynamic system, a diverse range of properties can be explored. Prominent examples of this are metastability (Bressler & Kelso, 2001; Deco, Jirsa, & McIntosh, 2011; Kelso, 1995; Tognoli & Kelso, 2009, 2014) and oscillations (Buzsáki, 2006; Buzsáki & Draguhn, 2004; Siegel, Donner, & Engel, 2012). Brain oscillations, inherently dynamic, have become a vital ingredient in proposed mechanisms ranging from psychological processes such as memory (Buzsáki, 2005; Friese et al., 2013; Montgomery & Buzsaki, 2007), and attention (Fries, 2005; Bosman et al., 2012), to basic neural communication in top-down or bottom-up information transfer (Bressler, Richter, Chen, & Ding, 2007; Buschman & Miller, 2007; Bastos et al., 2012; Bastos et al., 2015; Richter, Thompson, Bosman, & Fries, 2016; Richter, Coppola, & Bressler, 2016; Michalareas et al., 2016; van Kerkoerle et al., 2014).

Recently, approaches to study brain connectomics and the dynamics of neuronal communication have started to merge. A significant amount of work has recently been carried out that aims to quantify dynamic fluctuations of network activity in the brain using fMRI (Allen et al., 2014; Hutchison et al., 2013; Zalesky, Fornito, Cocchi, Gollo, & Breakspear, 2014; Shine et al., 2015; Thompson & Fransson, 2015a, 2016a; Shine, Koyejo, & Poldrack, 2016) as well as MEG (de Pasquale et al., 2010; de Pasquale et al., 2012; Hipp, Hawellek, Corbetta, Siegel & Engel, 2012; Baker et al., 2014; Michalareas et al., 2016). This research area which aims to unify brain connectommics with the dynamic properties of neuronal communication, has been called the “dynome” (Kopell, Gritton, Whittington, & Kramer, 2014) and the “chronnectome” (Calhoun, Miller, Pearlson, & Adalı, 2014). Since the brain can quickly fluctuate between different tasks, the overarching aim of this area of research is to understand the dynamic interplay of the brain’s networks. The intent of this research is that it will yield insight into the complex and dynamic nature of cognitive human abilities.

Although temporal network theory has been successfully applied in others fields (e.g., the social sciences), its implementation in network neuroscience has been limited. In the Theory and Methods section, we provide an introduction to temporal network theory, by extending the definitions and logic of static network theory, and define a selection of temporal network measures. In the Results section, we apply these measures to a resting-state fMRI dataset acquired during eyes-open and eyes-closed conditions, revealing differences in dynamic brain connectivity between subjects and conditions. Together this material illustrates the potential and varying information available from applying temporal network theory to network neuroscience.

## Theory and Methods

### From Static Networks to Temporal Networks

We begin by defining temporal networks, by expanding upon the basic definitions of network theory. In network theory, a graph (*G*) is defined as a set of nodes and edges:

G=(V,E), (1)

where V is a set containing *N* nodes. E is a set of tuples that represent the edges or connections between pairs of nodes (i,j):i,j∈V. The graph may have binary edges (i.e., an edge is either present or absent), or E may be weighted, often normalized between 0 and 1, to represent the magnitude of connectivity. When each edge has a weight, the definition of E is extended to a 3-tuple (*i*,*j*,*w*), where *w* denotes the weight of the edge between *i* and *j*. E is often represented as a matrix of the tuples, which is called a *connectivity matrix*, A (sometimes the term *adjacency matrix* is used). An element of the connectivity matrix *A*_*i*,*j*_ represents the degree of connectivity for a given edge. When *G* is binary, *A*_*i*,*j*_ = {0,1}, and in the weighted version, *A*_*i*,*j*_ = *w*. In the case of *A*_*i*,*j*_ = *A*_*j*,*i*_, for all *i* and *j*, the matrix is considered *undirected*; when this is not the case, the matrix is *directed*. With static networks, many different properties regarding the patterns of connectivity between nodes can be quantified, for example through centrality measures, hub detection, small-world properties, clustering, and efficiency (see Newman, 2010; Sporns, 2009; Bullmore & Sporns, 2009, for detailed discussion).

A graph is only a representation of some state of the world being modeled. The correspondence between the graph model and the state of the world may decrease due to aggregations, simplifications, and generalizations. Adding more information to the way that nodes are connected can entail that *G* provides a better representation, thus increasing the likelihood that subsequently derived properties of the graph will correspond with the state of the world being modeled. One simplification in Eq. 1 is that two nodes can be connected by one edge only.

To capture such additional information in the graph, edges need to be expressed along additional, nonnodal dimensions. We can modify Eq. 1 to

G=(V,E,D), (2)

where D is a set of the additional, nonnodal dimensions. In the case of multiple additional dimensions, D is a class of disjoint sets where each dimension is a set. Equation 2 is sometimes referred to in mathematics as a multigraph network, and in network theory as a *multilayer network* (Kivelä et al., 2014). For example, D could be a set containing three experimental paradigms {“*n* back,” “go/no go,” “Stroop task”} and/or temporal indices in seconds {0,1,2, … *T*}. A graph is said to be a temporal network when *D* contains an ordered set of temporal indices that represents time. This type of multilayer network is sometimes called a *multiplex* (Kivelä et al., 2014).

In a static graph, the edges E are elements that contain indices (2-tuples) for the nodes that are connected. In a multilayer network, E consists of (|D|+2)-tuples for binary graphs, where |D| expresses the number of sets in D. If time is the only dimension in D, then an element in E is a triplet (i,j,t):i,j∈V,t∈D. When *G* is weighted, E contains $\left((2\times |D|)+2\right)$-tuples as *w* becomes the size of D, representing one weight per edge.

While shorter definitions of temporal graphs are often used without using general definitions of multigraph/multilayer networks instead starting directly with the multiplex (see e.g., Holme & Saramäki, 2012; Masuda & Lambiotte, 2016), these formulations are mathematically equivalent when *D* only contains temporal information in Eq. 2. We have chosen to define a temporal network in terms of a multilayer network because this is appropriate when considering what a detailed network description of the human connectome will require. A multilayer network representation of the connectome could include many dimensions of information about an edge—for example, (i) activity 100 ms after stimulus onset (time), (ii) activity in the gamma frequency band, and (iii) activity associated with an *n*-back trial (task context). Thus we have introduced temporal networks by extending static network definitions to the broader concept of multilayer networks. However, in more complex multilayer networks with additional, nontemporal dimensions, the temporal-network measures presented in this article can be used to examine relationships across time, but either fixation or aggregation over the other dimensions will be required. However, more complex measures that consider all dimensions in *D* have been proposed elsewhere (e.g., Berlingerio, Coscia, Giannotti, Monreale, & Pedreschi, 2011).

For the remainder of this article we will consider only the case in which D contains only an ordered set of temporal indexes. In this case, each edge is indexed by *i*,*j* and *t*. To facilitate readability, connectivity matrix elements are written as $A_{i,j}^{t}$—that is, with the temporal index of D in the superscript. Instead of referring to “*A*^*t*^” as the “connectivity matrix at time point *t*”, some refer to this as a *graphlet* (Basu, Bar-noy, Johnson, & Ramanathan, 2010; Thompson & Fransson, 2015a, 2016a); others prefer to call this a *snapshot representation* (Masuda & Lambiotte, 2016), and still others call it a *supra-adjacency matrix* (Kivelä et al., 2014). It should be noted that some dislike the term *graphlet* due to possible confusion with the static network theory conception of a graphlet. Here a graphlet is a complete, independent two-dimensional connectivity matrix, but each graphlet is only a part of the entire network. Because graphlets do not need to be snapshots of temporal information under this definition, it is useful to describe what type of graphlet is being used. For example, a graphlet that expresses temporal information can be called a *time-graphlet* or *t-graphlet* (Thompson & Fransson, 2016a), and a graphlet carrying frequency information, an *f-graphlet* (Thompson & Fransson, 2015a).

Instead of representing the data with multiple graphlets, E can be used to derive the *contact sequence* or *event-based* representation containing the nodes and temporal index (Holme & Saramaäki, 2012; Masuda & Lambiotte, 2016). Unlike the graphlet representations, which must be discrete, contact sequences can also be used on continuous data and, when connections are sparse, can be a more memory-efficient way to store the data.

## Measures for temporal networks

Once the t-graphlets have been derived, various measures can be implemented in order to quantify the degree and characteristics of the temporal flow of information through the network. We begin by introducing two concepts that are used in several of the temporal network measures that will be defined later. The focus is on measures that derive temporal properties at a local level (i.e., per node or edge) or a global level (see Discussion for other approaches). We have limited our scope to describe only the case of binary, undirected, and discrete t-graphlets, although many measures can be extended to continuous time, directed edges, and nonbinary data.

### Concept: Shortest Temporal Path

In static networks, the *shortest path* is the minimum number of edges (or sum of edge weights) that it takes for a node to communicate with another node. In temporal networks, a similar measure can be derived. Within temporal networks, we can quantify the time taken for one node to communicate with another node. This is sometimes called the “shortest temporal distance” or “waiting time.” Temporal paths can be measured differently by calculating either how many edges are traveled or how many time steps are taken (see Masuda & Lambiotte, 2016); here we quantify the time steps taken.

**Figure 1.**
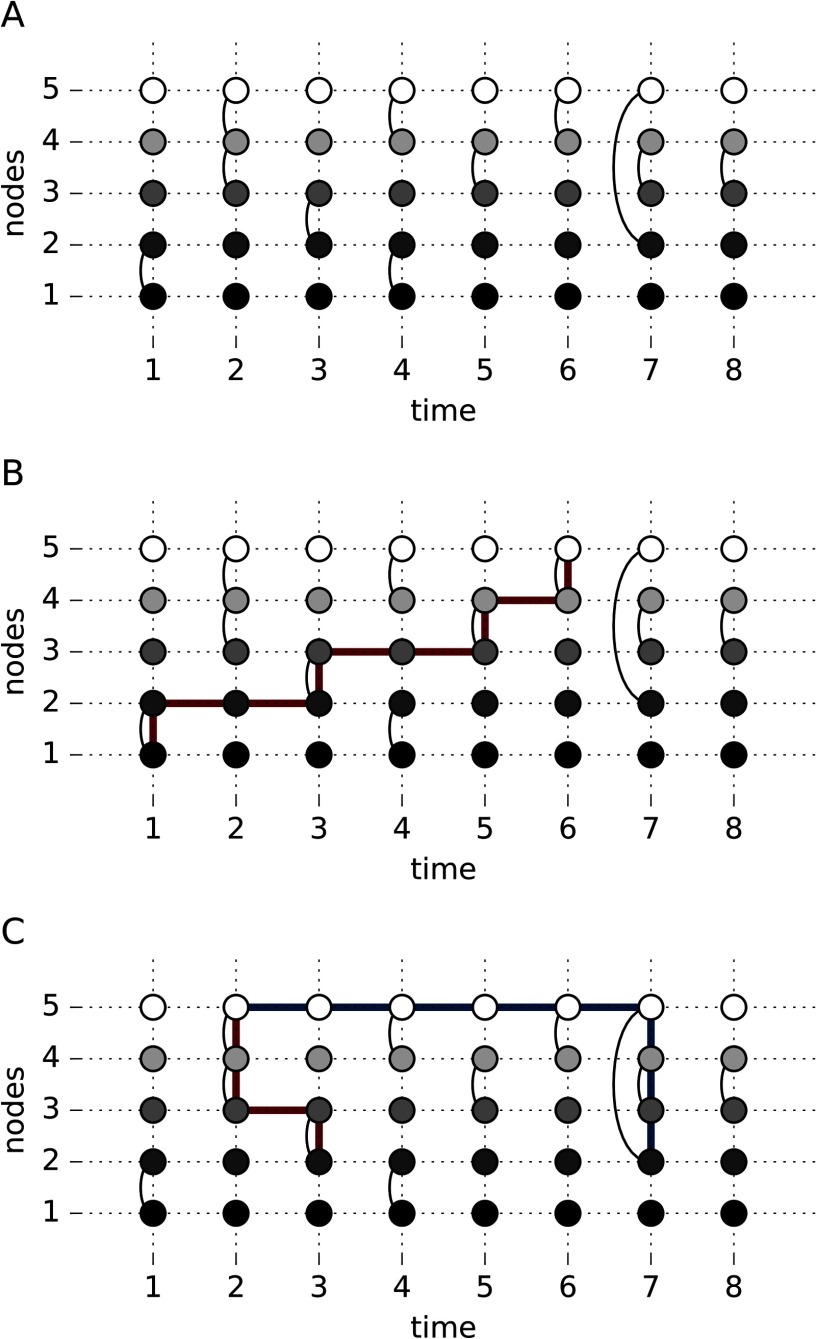
Illustration of the concept of the shortest temporal path. (A) The basic layout of a temporal network, viewed in a slice graph representation. (B) The red line indicates the shortest temporal path possible for Node 1 to reach Node 5. (C) The difference in shortest paths that arises when a single (blue line) or multiple (red line) edges can be traveled at a single time point.

Consider the temporal network shown in Figure 1A. Starting at Time Point 1, the shortest temporal path for Node 1 to reach Node 5 is five time units (Figure 1B, red line). Shortest temporal paths can never travel edges from a previous time point (i.e., backward in time), but it is possible for multiple edges to be visited at each time step. It is thus necessary to determine how many edges can be traveled at each time point. For example, Node 5 at Time Point 2 can reach Node 3 in one time step if we allow multiple edges to be traveled (Figure 1C, red line). If multiple edges cannot be traveled, then the shortest path for Node 5 to reach Node 2, starting at Time Point 2, is five time units (Figure 1C, blue line). Thus, a parameter must be set that restrains how many edges per time point can be traveled. This parameter should depend on the temporal resolution of the data and is best chosen given previously established knowledge of the dynamics of the data. For fMRI, where the temporal resolution is in seconds, it makes sense to assume that several edges can be traveled per unit of time. Contrarily, in MEG, where the resolution is in the range of milliseconds, it is more reasonable to place a limit on the number of edges that can be traveled per time unit.

Regarding the shortest temporal path, it is useful to keep in mind that the path is rarely symmetric, not even when the t-graphlets themselves are symmetric. This is illustrated by considering the network shown in Figure 1A, in which it takes five units of time for Node 1 to reach Node 5 when starting at *t* = 1. However, for the reversed path, it takes only three units of time for Node 5 to reach Node 1 (allowing for multiple edges to be traveled per time point).

Some consideration is needed of whether thinking about shortest temporal paths makes sense in all neuroimaging contexts. Shortest temporal paths represent information being transferred in a network. Thus, the concept of the shortest temporal path is appropriate in the situation in which one can assume that the transfer of information is continued across subsequent time points. When the time scale is in the range of milliseconds (e.g., EEG and MEG), the shortest temporal path should be unproblematic to interpret. In contrast, for a longitudinal study in which the temporal resolution is in years, the concept of the shortest temporal path makes no sense. Less clear-cut situations are neuroimaging techniques with sluggish temporal resolution (e.g., fMRI). However, the shortest temporal path seems to be a reasonable measure for ongoing BOLD signals that allow for considering previously observed temporal dynamics, including avalanche behavior (Tagliazucchi, Balenzuela, Fraiman, & Chialvo, 2012), bursty behavior (Thompson & Fransson, 2016a), and metastability (Deco & Kringelbach, 2016).

### Concept: Intercontact Time

The intercontact time between two nodes is defined as the temporal difference distinguishing two consecutive nonzero edges between those nodes. This definition differs from the shortest temporal path in so far as it only considers direct connections between two nodes. Considering Figure 1A, the intercontact times between Nodes 4 and 5 become a list [2,2], since there are edges present at Time Points 2, 4, and 6. Each edge will have a list of intercontact times. The number of intercontact times in each list will be the number of nonzero edges between the nodes minus one. Unlike with shortest temporal paths, graphs that contain intercontact times will always be symmetric.

### Nodal Measure: Temporal Centrality

A node’s influence in a temporal network can be calculated in a way akin to degree centrality in the static case, where the sum of the edges for a node is calculated. The difference from its static counterpart is that we also sum the number of edges across time. Formally, the temporal degree centrality, *D*^*T*^, for a node *i* is computed as

$$D_{i}^{T}=\overset{N}{\underset{j=1}{\sum }}\overset{T}{\underset{t=1}{\sum }}A_{i,j}^{t},$$ (3)

where *T* is the number of time points, *N* is the number of nodes, and $A_{i,j}^{t}$ is a graphlet.

**Figure 2.**
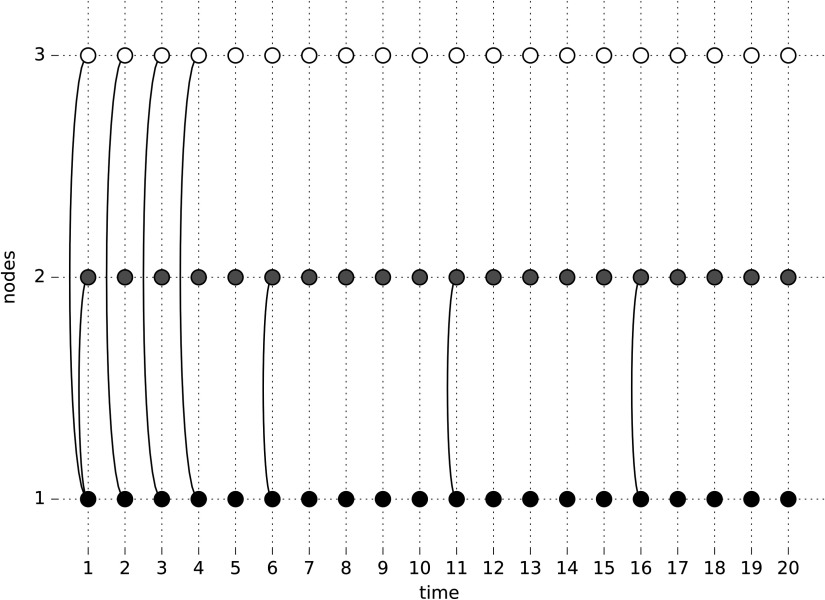
A slice graph representation of a simple example of a temporal network that illustrates the conceptual difference between temporal degree centrality and temporal closeness centrality.

While it provides an estimate of how active or central a node is in a temporal network, temporal degree centrality does not quantify the temporal order of the edges. This is illustrated in Figure 2, where Node 3 and Node 2 have identical temporal degree centralities, despite having very different temporal ordering of their edges.

### Nodal Measure: Temporal Closeness Centrality

A centrality measure that does consider the temporal order is temporal closeness centrality (Pan & Saramäki, 2011). This is an extension of the static closeness centrality, which is the inverse sum of the shortest paths. Temporal closeness centrality is calculated as

$$C_{i,t}^{T}=\frac{1}{N-1}\overset{N}{\underset{j=1}{\sum }}\frac{1}{d_{i,j}^{\tau }},$$ (4)

where *d*_*i*,*j*_^*τ*^ is the average shortest path between nodes *i* and *j* across all time points for which a shortest path exists. As in the static counterpart, if a node has shorter temporal paths than other nodes, it will have a larger temporal closeness centrality.

Consider the example given in Figure 2, which shows a temporal sequence of connectivity among three nodes over 20 time points. Note that the temporal degree centralities are identical for both Node 2 and Node 3, while the degree centrality for Node 1 is twice as large. Node 2 has the largest temporal closeness centrality, since the time between edges is longer for Node 2 than for Node 3, which has the lowest value of temporal closeness centrality.

### Edge Measure: Bursts

By using temporal network theory, bursts have been identified as an important property of many processes in nature (Barabási, 2005, 2010; Vázquez et al., 2006; Vazquez, Rácz, Lukács, & Barabási, 2007; Min, Goh, & Vazquez, 2011). A hallmark of a bursty edge is the presence of multiple edges with short intercontact times, followed by longer and varying intercontact times. In statistical terms, such a process is characterized by a heavy-tailed distribution of intercontact time probabilities. Numerous patterns of social communication and behavior have been successfully modeled as bursty in temporal network theory, including email communication (Barabási, 2005; Eckmann, Moses, & Sergi, 2004), mobile phone communication (Jo, Karsai, Kertész, & Kaski, 2012), spreading of sexually transmitted diseases (Vazquez, 2013), soliciting online prostitution (Rocha, Liljeros, & Holme, 2010), and epidemics Takaguchi, Masuda, & Holme, 2013. With regard to network neuroscience, we have recently shown that bursts of brain connectivity can be detected in resting-state fMRI data (Thompson & Fransson, 2016a). Furthermore, bursty temporal patterns have also been identified in the amplitudes of the EEG alpha signal (Freyer, Aquino, Robinson, Ritter, & Breakspear, 2009; Freyer, Roberts, Ritter, & Breakspear, 2012; Roberts, Boonstra, & Breakspear, 2015).

There are several strategies to quantify bursts. A first way to check whether a time series of brain connectivity between two nodes is bursty is simply to plot the distribution of intercontact times. Thus, the complete distribution of *τ* for a given edge contains information about the temporal evolution of brain connectivity. However, other methods are available to quantify bursts. One example is the burstiness coefficient (*B*), first presented in Goh and Barabási (2008) and formulated for discrete graphs, Holme and Saramäki (2012):

$$B_{ij}=\frac{\sigma (\tau_{ij})-\mu (\tau_{i,j})}{\sigma (\tau_{ij})+\mu (\tau_{ij})},$$ (5)

where *τ*_*ij*_ is a vector of the intercontact times between nodes *i* and *j* through time. When *B* > 0, it is an indication that the temporal connectivity is bursty. This occurs when the standard deviation *σ*(*τ*) is greater than the mean *μ*(*τ*). In Eq. 5, bursts are calculated per edge, which can be problematic when only a limited number of data are available. Functional imaging sessions must be long enough to accurately establish whether or not a given temporal distribution is bursty (too few intercontact times will entail too poor an estimation of *σ* to accurately estimate *B*). Typically, for resting-state fMRI datasets acquired over rather short time spans (5–6 minutes) with low temporal resolution (typically 2–3 seconds), it might be difficult to quantify *B* in a single subject. A potential remedy in some situations is to compute *B* after concatenating intercontact times across subjects.

Equation 5 calculates the number of bursts per edge. This can easily be extended to a nodal measure by summing over the bursty coefficients across all edges for a given node. Alternatively, a nodal form of *B* can be calculated by using the intercontact times for all *j* instead of averaging over *j* in *B*_*ij*_. Finally, if a process is known to be bursty, instead of quantifying *B*, it is possible simply to count the number of bursts present in a time series.

### Global Measure: Fluctuability

Although centrality measures provide information about the degree of temporal connectivity, and bursts describe the distribution of the temporal patterns of connectivity at a nodal level, one might also want to retrieve information about the global state of a temporal network. To this end, *fluctuability* aims to quantify the temporal variability of connectivity. We define the fluctuability *F* as the ratio of the number of edges present in *A* over the grand sum of *A*_*t*_:

$$F=\frac{\underset{i}{\sum }\underset{j}{\sum }U(A_{i,j})}{\underset{i}{\sum }\underset{j}{\sum }\underset{t}{\sum }A_{i,j}^{t}},$$ (6)

where *U* is a function that delivers a binary output. *U*(*A*_*i*,*j*_)is set to 1 if at least one of an edge occurs between nodes *i* and *j* across times *t* = 1, 2, …, *T*. If not, *U*(*A*_*i*,*j*_)is set to 0. This can be expressed as

$$U(A_{ij})=\left\{\begin{array}{ll} 1 & \text{if}\;\overset{T}{\underset{t}{\sum }}A_{ij}^{t}>0,\\ 0 & \text{if}\;\overset{T}{\underset{t}{\sum }}A_{ij}^{t}=0, \end{array}\right.$$ (7)

where *T* is the total number of time points and *A* has at least one nonzero edge. From the definition given in Eq. 6, it follows that the maximum value of *F* is 1 and that this value only occurs when every edge is unique and occurs only once in time.

**Figure 3.**
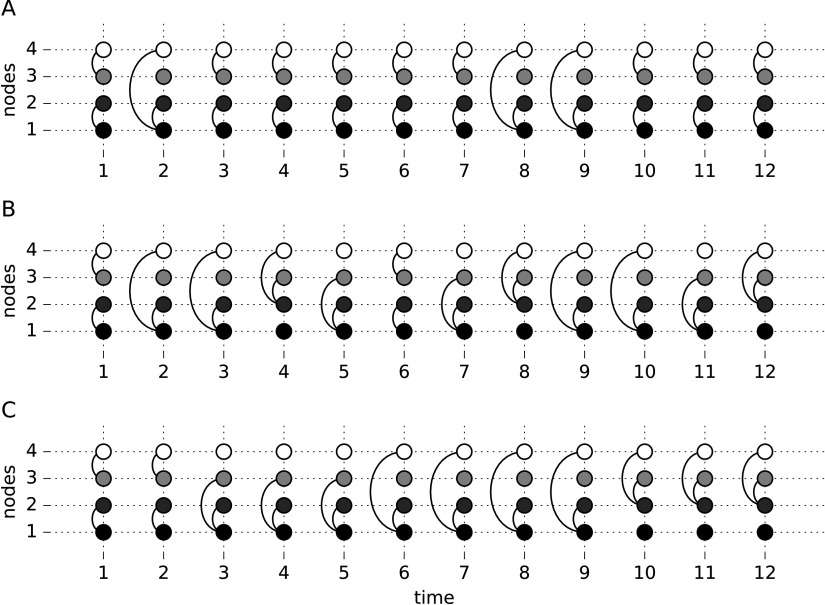
Illustration of the fluctuability and volatility measures. The temporal networks shown in panels A, B, and C all have the same numbers of nodes and edges, but they differ in fluctuability and volatility. (A) This network has low fluctuability (*F* = 0.125) and volatility (*V* = 0.73). (B) This network has the highest volatility (*V* = 2.55) of all three networks and a fluctuability (*F* = 0.25) equal to that of the network in panel C. (C) This network has lower volatility than B (*V* = 1.27) but equal fluctuability (*F* = 0.25).

While the above definition of fluctuability may seem counterintuitive, it is an adequate measure to quantify the temporal diversity of edges. *F* reveals how connectivity patterns within the network fluctuate across time. To see this, consider the networks shown in Figures 3A and 3B, for which two edges are present at each time point. There are only three unique edges in Figure 3A, meaning that the sum of *U* is 3 for the network shown in Figure 3A. However, there is greater fluctuation in edge configuration for the network shown in Figure 3B, and all six possible edges are present (entailing that the sum of *U* is equal to 6). Since both networks have in total 24 connections over time, it becomes easy to see that the network shown in Figure 3B has twice as large a value of *F* as the network shown in Figure 3A.

Notably, fluctuability is insensitive to the temporal order of connectivity. For example, the networks depicted in Figures 3B and 3C have the same fluctuability, despite having considerably different temporal orders of edge connectivity. Thus, fluctuability can be used as an indicator of the overall degree of spatial diversity of connectivity over time.

The definition of fluctuability can be changed to work at a nodal level. To achieve this, the summation in Eq. 6 is applied over only one of the nodal dimensions. Note that for nodes with no connections at all, the denominator will be 0, and to circumvent this hindrance, the nodal fluctuability $F_{i}^{N}$ is defined as

$$F_{i}^{N}=\left\{\begin{array}{ll} \frac{\underset{j}{\sum }U(A_{i,j})}{\underset{j}{\sum }\underset{t}{\sum }A_{i,j}^{t}} & \text{if}U(A_{i,j})>0,\\ 0 & \text{if}U(A_{i,j})=0. \end{array}\right.$$ (8)

### Global Measure: Volatility

One possible global measure of temporal order is how much, on average, the connectivity between consecutive t-graphlets changes. This indicates how volatile the temporal network is over time. Thus, volatility (*V*) can be defined as

$$V=\frac{1}{T-1}\overset{T-1}{\underset{t=1}{\sum }}D(A^{t},A^{t+1}),$$ (9)

where *D* is a distance function and *T* is the total number of time points. The distance function quantifies the difference between a graphlet at *t* and the graphlet at *t* + 1. In all the following examples in this article, for volatility we use the Hamming distance, because it is appropriate for binary data.

Whereas there was no difference in fluctuability between the networks shown in Figures 3B and 3C, there is a difference in volatility, since the network in Figure 3B has more abrupt changes in connectivity than the network shown in Figure 3C.

Extensions of the volatility measure are possible. Like fluctuability, volatility can be defined at a local level. A per-edge version of volatility can be formulated as

$$V_{i,j}^{L}=\frac{1}{T-1}\overset{T-1}{\underset{t=1}{\sum }}D(A_{i,j}^{t},A_{i,j}^{t+1}).$$ (10)

Additionally, taking the mean $V_{i,j}^{L}$ over *j* would give an estimate of volatility centrality.

### Global Measure: Reachability Latency

Measures of reachability focus on estimating the time taken to “reach” the nodes in a temporal network. In Holme (2005), both the *reachability ratio* and *reachability time* are used. The reachability ratio is the percentage of edges that have a temporal path connecting them. The reachability time is the average length of all temporal paths. However, when applying reachability to the brain, the two aforementioned measures are not ideal, given the noncontroversial assumption that any region in the brain, given sufficient time, can reach all other regions.

With this assumption in mind, we define a measure of reachability, *reachability latency*, that quantifies the average time it takes for a temporal network to reach an a-priori-defined reachability ratio. This is defined as

$$R_{r}=\frac{1}{TN}\underset{t}{\sum }\underset{i}{\sum }d_{i,k}^{t},$$ (11)

where $d_{i}^{t}$ is an ordered vector of length *N* of the shortest temporal paths for node *i* at time point *t*. The value *k* represents the ⌊*rN*⌉th element in the vector, which is the rounded product of the fraction of nodes that can be reached, *r*, with *N* being the total number of nodes in the network.

In the case of *r* = 1, (i.e., 100% of nodes are reachable), Eq. 11 can be rewritten as

$$R_{1}=\frac{1}{TN}\underset{t}{\sum }\underset{i}{\sum }\underset{j}{\max}d_{i,j}^{t}.$$ (12)

Equation 12 has been referred to as the *temporal diameter* of the network (Nicosia et al., 2013). If Eq. 12 were modified and calculated per node instead of averaging over nodes, it would be a temporal extension of node eccentricity.

Unless all nodes are reached at the last time point in the sequence of recorded data, there will be a percentage of time points from which all nodes can not be reached. This effectively reduces their value of *R*, because $d_{i,j}^{t}$ cannot always be calculated, but *R* is still normalized by *T*. If this penalization is considered too unfair, it is possible to normalize *R* by replacing *T* with T*, which is the number of time points where $d_{i,j}^{t}$ has a real value.

### Global Measure: Temporal Efficiency

A similar concept is the idea of temporal efficiency. In the static case, efficiency is computed as the inverse of the average shortest path for all nodes. Temporal efficiency is first calculated at each time point as the inverse of the average shortest path length for all nodes. Subsequently, the inverse average shortest path lengths are averaged across time points to obtain an estimate of global temporal efficiency, which is defined as

$$E=\frac{1}{T(N^{2}-N)}\underset{i,j,t}{\sum }\frac{1}{d_{i,j}^{t}},i\neq j.$$ (13)

Although reachability and efficiency estimate similar temporal properties, since both are based on the shortest temporal paths, the global temporal efficiency may result in different results than the reachability latency. This is because efficiency is proportional to the average shortest temporal path, whereas reachability is proportional to the longest shortest temporal path to reach *r* percent of the network. Similar to the case of static graphs, temporal efficiency could be calculated at a nodal level as well, and this would equal the closeness centrality.

### Summary of Temporal Network Measures

In Table 1 we provide a brief summary of the temporal network measures outlined here, accompanied by short descriptions. We also signify which measures are sensitive to temporal order.

**Table 1.** Summary of the temporal network measures outlined in this article

**Measure**	**Description**	**Order Dependent?**	**Variable**
Temporal centrality	Number of overall connections in time	N	*D*^*T*^
Closeness centrality	Time between connections	Y	*C*^*T*^
Burstiness	Distribution of subsequent connections	Y	*B*_*ij*_
Fluctuability	Ratio of unique edges vs. all edges	N	*F*
Volatility	Rate of change of graphlets per time point	Y	*V*
Reachability latency	Time taken for all nodes to be able to reach each other	Y	*R*_*r*_
Temporal efficiency	Inverse average shortest temporal path	Y	*E*

### Statistical Considerations of Temporal Network Measures

When implementing temporal graph measures, it is important to perform adequate statistical tests to infer differences between the subject groups, task conditions, or chance levels. For group comparisons, nonparametric permutation methods are advantageous where the group assignment of the calculated measure can be shuffled between the groups and a null distribution can be created. Alternatively, to justify that a measure is significantly present above chance levels, the construction of null graphs is required. There are multiple ways to create temporal null graphs, and they each have their own benefits and drawbacks. One method is to permute the temporal order of entire time series, but this will destroy any autocorrelation present in the data. Another alternative is to permute the phase of the time series prior to thresholding the t-graphlets. A third option would be to permute blocks of time series data, but this may not be appropriate for all network measures (e.g., volatility). A fourth option would be to use vector autoregressive null models (Chang & Glover, 2010; Zalesky et al., 2014). We refer the reader to Holme & Saramäki (2012) for a full account of approaches to performing statistical tests on measures derived from temporal network theory.

## Methods

### fMRI data

Two resting-state fMRI sessions (3-tesla, TR = 2,000 ms, TE = 30 ms) from 48 healthy subjects were used in the analysis (19–31 years, 24 female). The fMRI data were downloaded from an online repository: the Beijing Eyes-Open/Eyes-Closed dataset, available at www.nitrc.org (Liu & Duyn, 2013). Each functional volume comprised 33 axial slices (thickness/gap = 3.5/ 0.7 mm, in-plane resolution = 64 × 64, field of view = 200 × 200 mm). The dataset contained three resting-state sessions per subject, and each session lasted 480 s (200 image volumes, two eyes-closed sessions and one eyes-open session). We used data only from the 2nd or 3rd session, which were the eyes-open (EO) and second eyes-closed (EC) sessions, where the order was counterbalanced across subjects. Two subjects were excluded due to incomplete data. Further details regarding the scanning procedure are given in Liu & Duyn (2013).

All resting-state fMRI data was preprocessed using Matlab (Version 2014b, Mathworks, Inc.) with the CONN (Whitfield-Gabrieli & Nieto-Castanon, 2012) and SPM8 (Friston et al., 1995) toolboxes. The functional imaging data were realigned and normalized to the EPI MNI template as implemented in SPM. Spatial smoothing was applied using a Gaussian filter kernel (FWHM = 8 mm). Additional image artifact regressors attributed to head movement (Dijk, Sabuncu, & Buckner, 2012; Power, Barnes, Snyder, Schlaggar, & Petersen, 2012) were derived by using the ART toolbox for scrubbing (www.nitrc.org). Signal contributions from white brain matter, cerebrospinal fluid (CSF), and head movement (six parameters), as well as the ART micromovement regressors for scrubbing, were regressed from the data using the CompCor algorithm (Behzadi, Restom, Liau, & Liu, 2007; the first five principal components were removed for both white matter and CSF). After regression, the data were band-passed between 0.008 and 0.1 Hz, as well as linearly detrended and despiked. Time series of fMRI brain activity were extracted from 264 regions of interest (ROIs; spherical with a 5-mm radius) using the parcellation scheme for cortex and subcortical structures described in Power et al. (2011). Each ROI was normalized by demeaning and scaling the standard deviation to 1. These 264 ROIs were further divided into ten brain networks, as described in Cole et al. (2013) (technically *subgraphs*, in network theory terminology). Automatic anatomical labeling (AAL) regions associated with specific ROIs, shown in the Supplementary Tables (Thompson, Brantefors, & Fransson, 2017), were determined by taking the AAL region at (or closest to) the center of the ROI. Note that this offers only an approximate anatomical labeling of the positions of ROIs.

### Creating Time-Graphlets (t-Graphlets)

While there are many proposed methods for dynamic functional connectivity (Smith et al., 2012; Allen et al., 2014; Liu & Duyn, 2013; Lindquist, Xu, Nebel, & Caffo, 2014; Shine et al., 2015; Thompson & Fransson, 2016a), we chose a weighted correlation strategy (described below) because it does not require optimizing any parameters or clustering. The method is based on our previous work (Thompson & Fransson, 2016a), using the same fundamental assumptions, which results in high temporal sensitivity to fluctuating connectivity. However, we here extended the method presented in Thompson & Fransson (2016a) so that it would compute unique connectivity estimates for each time point, and thereby avoid the necessity to cluster the data using a clustering technique such as *k*-means.

Our logic was to calculate dynamic functional brain connectivity estimates based on a weighted Pearson correlation. To calculate the conventional Pearson correlation coefficient, all points are weighted equally. In the weighted version, data points contribute differently to the correlation coefficient, depending on what weight they have been assigned. These weights are then used to calculate the weighted mean and weighted covariance to estimate the weighted correlation coefficient. By using a unique weighting vector per time point, we were able to get unique connectivity estimates for each time point.

The weighted Pearson correlation between the signals *x* and *y* is defined as

$$r(x,y;w)=\frac{\Sigma_{x,y;w}}{\Sigma_{x,x;w}\Sigma_{y,y;w}},$$ (14)

where **Σ** is the weighted covariance matrix and *w* is a vector of weights that is equal in length to *x* and *y*. The weighted covariance matrix is defined as

$$\Sigma_{x,y;w}=\frac{\overset{n}{\underset{i}{\sum }}w_{i}(x-\mu_{x;w})(y-\mu_{y;w})}{\overset{n}{\underset{i}{\sum }}w_{i}},$$ (15)

where *n* is the length of the time series. Note that **Σ** is the covariance matrix and $\sum_{i}^{n}$ is a sum over time points. The variables *μ*_*x*;*w*_ and *μ*_*y*;*w*_ are the weighted means, defined as

$$\mu_{x;w}=\frac{\overset{n}{\underset{i}{\sum }}w_{i}x_{i}}{\overset{n}{\underset{i}{\sum }}w_{i}},\mu_{y;w}=\frac{\overset{n}{\underset{i}{\sum }}w_{i}y_{i}}{\overset{n}{\underset{i}{\sum }}w_{i}}.$$ (16)

Equations 14–16 define the weighted Pearson coefficient with the exception of the weight vector *w*. If every element in *w* is identical, we can easily observe that the unweighted (conventional) Pearson coefficient will be calculated. Here, we instead wished to calculate a unique *w* for each time point, providing a connectivity estimate based on the weighted mean and weighted covariance.

Different weighting schemes could be applied. In fact, many of the different dynamic connectivity methods proposed in the literature are merely different weighting schemes (e.g., a nontapered sliding window approach is just a binary weight vector).

We decided upon a global weighting of the spatial dimensions by calculating the distance between the nodes at a specific time point with all other nodes for every other time point. This entails that the weights for the covariance estimates at *t* are larger for other time points that display a global spatial pattern across all nodes similar to that of the nodes at *t*. A new weight vector is calculated for each time point. The unique weight vector per time point produces a unique weighted Pearson correlation at each time point. This reflects the weighted covariance, where time points with similar global spatial brain activation are weighted higher. This produces, for each edge, a connectivity time series with fluctuating covariance.

More formally, the weights for estimating the connectivity at time *t* are derived by taking the distance between the activation of the ROIs at *t* and at each other time point (indexed by *v*):

$$w_{v}^{t}=\frac{1}{D(y_{t},y_{v})},$$ (17)

where *D* is a distance function and *y* is the multivariate time series of the ROIs. For the distance function, we used Euclidean distance (i.e., $D(a,b)=\sqrt{\left[\sum_{i}^{n}{\left(a_{i}-b_{i}\right)}^{2}\right]}$).

**Figure 4.**
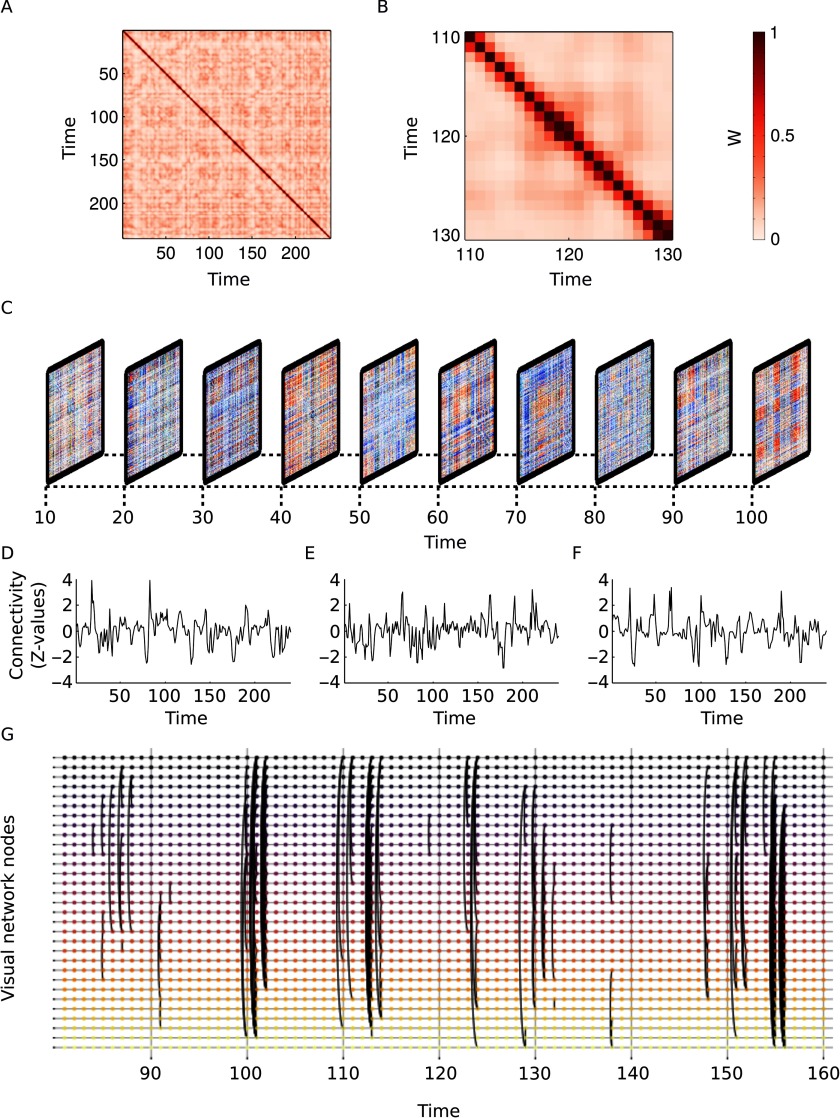
Outline of the weighted correlation method to estimate dynamic connectivity. (A) Collection of weight vectors for a single subject in the eyes-open condition. (B) Collection of weight vectors for a single subject in the eyes-closed condition. (C) A magnified portion of the image shown in panel A that highlights that neighboring time points have the highest weights. (D) Stack of graphlets showing the weighted connectivity for a selection of the resting-state session. (E–G) Example connectivity time series for the entire session for a single edge. The edges are (E) two nodes from the default mode network, (F) two nodes in the visual network, and (G) one node in the visual network and one node in the default mode network. (H) Example of the slice graph representation of temporal brain connectivity for all nodes in the visual subnetwork with binary connections. All panels in this figure show data taken from the same subject and, with the exception of panel B, show the EO condition. All time units in the figure are given in volumes (TR) (i.e., each time step is 2 s).

The weight vector of *t* is created by applying Eq. 17 for all *v* ∈ *T*,*v* ≠ *t*. This implies that at the time point of interest, *t*, we calculate a vector of weights (indexed by *v*) that reflects how much the global spatial pattern of brain activity (i.e., all ROIs) differs from the brain activity at *t*. Each collection of weight vectors *w*^*t*^ can form a *t* by *t* matrix **w** for each subject and each condition. The values of each matrix are scaled to range between 0 and 1. Finally, the diagonal of **w** is set to 1. The collection of weight vectors for a single subject is shown for both EO and EC sessions in Figures 4A–4C. Although this is not explicitly assumed in our method, neighboring time points have the highest weights (Figure 4C).

After the derivation of the connectivity time series, a Fisher transform and a Box–Cox transform were applied. For the Box–Cox transform, the *λ* parameter was fit by taking the maximum likelihood after a grid-search procedure from -5 to 5 in increments of 0.1 for each edge. Prior to the Box–Cox transformation, the smallest value was scaled to 1 to make sure the Box–Cox transform performed similarly throughout the time series (Thompson & Fransson, 2016b). Each connectivity time series was then standardized by subtracting the mean and dividing by the standard deviation. Snapshots of the weighted graphlets can be seen in Figure 4D. The entire connectivity time series for three different ROI pairings are shown in Figures 4E–4G. Binary t-graphlets were created by setting edges exceeding two standard deviations to 1, or otherwise 0, for each time series.

Our thresholding approach to create binary connectivity matrices is suboptimal and could be improved upon in future work (see Discussion). The need to formulate more robust thresholding practices has been an ongoing area of research in static network theory in the neurosciences (Drakesmith et al., 2015). Similar work needs to be carried out for temporal networks, because a limitation of the current approach is a heightened risk of false positive connections.

### Tools for Temporal Network Theory

We have implemented all temporal network measures described in the present work in a Python package of temporal network tools called Teneto (www.github.com/wiheto/teneto) for Python 3.x, although the package itself is still under development. The package currently contains code for all the measures mentioned above and plotting functions for slice plots (e.g., Figure 4F) and for stacking graphlets (e.g., Figure 4D). Data formats for both the graphlet/ snapshot and event/contact sequence data representations are available.

### Statistics

All between-group comparisons in the next section use the between-group permutation method outlined previously. Null distributions were created with 100,000 permutations of shuffling which group each subject’s EO/EC results belonged to, and all comparisons were two tailed. For between-subjects comparisons, Spearman rank correlations were used. To determine which nodes had a higher-than-chance level of centrality, 1,000 permutations were performed in which the nodal order for each subject was shuffled. This resulted in 264 null distributions in which the centrality was averaged over subjects. The distribution with the largest 950th value was selected to signify *p* = 0.05.

## Results

### Applying Temporal Degree Centrality and Temporal Closeness Centrality

With temporal centrality measures we can formulate research questions along the following lines: (i) which nodes have the most connections through time (temporal degree centrality), or (ii) which nodes have short temporal paths to all other nodes (temporal closeness centrality). For the shortest-paths calculations, we allowed all possible steps at a single time point to be used in this example.

**Figure 5.**
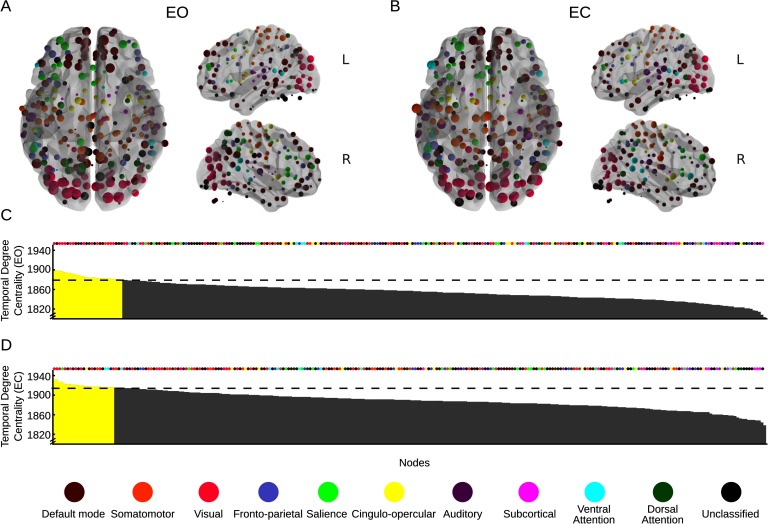
Spatial patterns of temporal degree centrality displayed on the surface of the brain for (A) the eyes-open (EO) condition and (B) the eyes-closed (EC) condition. (C) Spatial distribution of temporal degree centrality across all nodes for the EO condition. Yellow color indicates *p* < 0.05. The assigned network of each node is marked by colored dots above the plot. (D) As in panel C, but for the EC condition. Anatomically detailed information about the nodes that had the largest degrees of temporal centrality in panels C and D can be found in Supplementary Tables 1 and 2 Thompson et al., 2017. Nodes located outside the rendered brain images are part of the cerebellum.

First we illustrate the spatial distribution of both centrality measures in the brain. Temporal degree centrality, averaged over all subjects, is displayed on the brain for all 264 ROIs for both the EO (Figure 5A) and EC (Figure 5B) conditions, respectively. Nodes with a higher-than-expected temporal centrality degree (*p* < 0.05) are shown for both conditions in Figures 5C and 5D, respectively. Tables of all nodes/brain regions that passed the significance threshold are presented in Supplementary Tables 1 and 2 (Thompson et al., 2017). Of the 25 nodes in the EO condition that were above the threshold, the majority were located in either the visual network (12 nodes) or the default mode network (eight nodes). In the EC condition, 26 nodes passed the statistical threshold. Somewhat surprisingly, many of these nodes still came from the visual network, but relatively fewer than in the EO condition (nine nodes). Speculatively, the relatively high centrality of nodes in the visual network might be related to the notion that many subjects may have performed mental imagery or other activity known to activate areas of visual cortex during the EC condition (Ganis, Thompson, & Kosslyn, 2004).

**Figure 6.**
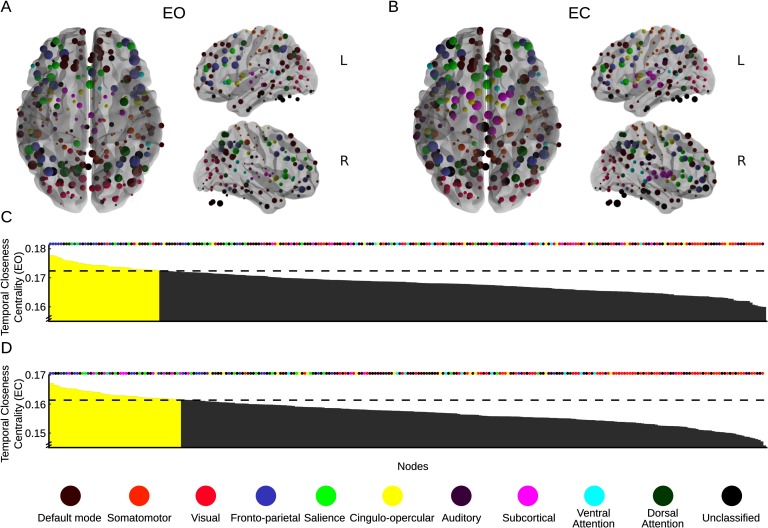
Spatial patterns of temporal closeness centrality displayed on the brain surface for the eyes-open (EO) condition (A) and the eyes-closed (EC) condition (B). (C) Distribution of temporal closeness degrees over all nodes for the EO condition. Yellow color indicates *p* < 0.05. The assigned network of each node is marked by colored dots above the plot. (D) As in panel C, but for the EC condition. Anatomically detailed information about the largest nodes in panels C and D with the greatest temporal closeness centrality can be found in Supplementary Tables 3 and 4 Thompson et al., 2017. Nodes outside the rendered brain images are part of the cerebellum.

For closeness centrality, we observed that in both the EO condition (Figure 6A) and the EC condition (Figure 6B), nodes with higher-than-expected temporal closeness centrality (*p* < 0.05) were located in the frontoparietal, dorsal attention, and default mode networks (Figures 6C and 6D) (see also Thompson et al., 2017, Supplementary Tables 3 and 4). Notably, for the EO condition only three nodes in the visual network had a closeness centrality above the threshold, whereas the EC condition had none. On the other hand, nodes in the saliency and subcortical networks scored higher in the EC than in the EO condition.

In sum, both centrality measures returned reasonable spatial distributions of high-scoring nodes across the brain for both conditions, implying that they quantified relevant and interesting information about the dynamics of the BOLD signal. Obviously, this demonstration is exploratory, and thus we are unable to infer the underlying cognitive processes from the given centrality measures alone. This task is also made more difficult by the cognitively unrestrained behavioral conditions in a resting state. For example, the high closeness centrality of the saliency network in the EC condition might depend on a number of factors, ranging from focus on the task at hand to the presence of emotional processes. However, the results allow us to consider how the different centrality measures can provide novel insights into the network dynamics. That the visual network has the highest centrality in the EO condition for both measures is reasonable. The observation that the default mode and attentional networks also score high on both centrality measures also seems reasonable. If, on the other hand, the somato-motor network had scored high in both EC and EO, or if the centrality of nodes in the visual network had been higher during EC than EO, such results would call into question whether our temporal centrality measures were actually quantifying anything meaningful.

**Figure 7.**
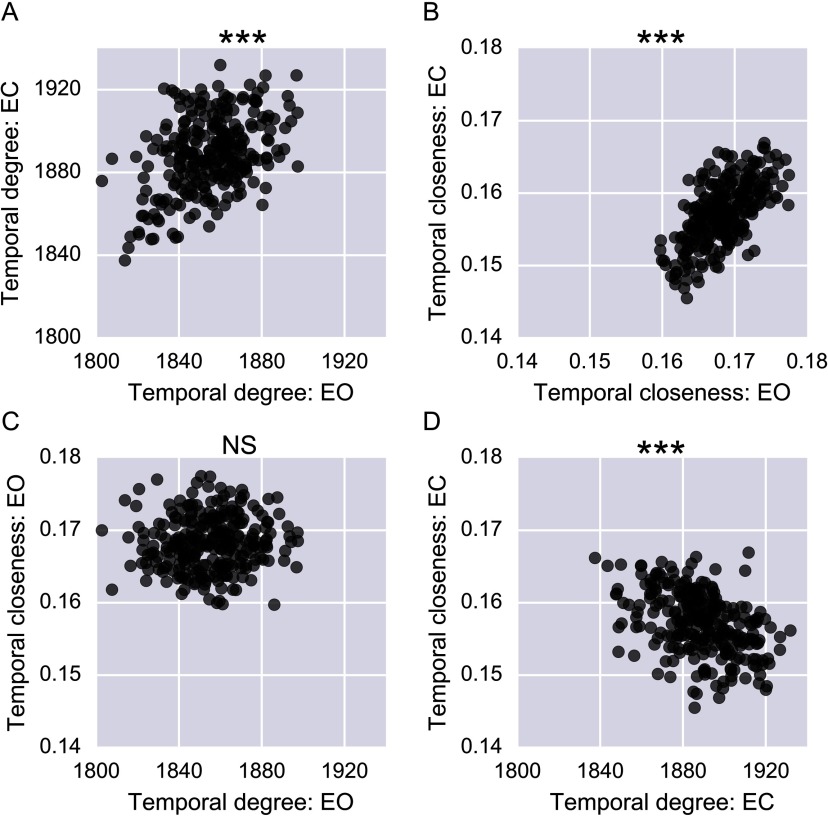
Applying temporal degree centrality and temporal closeness centrality for the eyes-open (EO) and eyes-closed (EC) conditions. Each dot represents the centrality of a node. (A) Temporal degree centrality for the EO versus the EC condition. (B) Temporal closeness centrality for the EO versus the EC condition. (C) Temporal degree centrality versus temporal closeness centrality in the EO condition. (D) Temporal degree versus temporal closeness centrality in the EC condition. *** signifies *p* < 0.001.

The centrality estimates for nodes were compared across imaging sessions to evaluate whether the temporal patterns were similar across subjects. Despite differences in the highest-scoring nodes for each condition, temporal degree centrality correlated significantly between the EO and EC conditions (Figure 7A, *ρ* = 0.35, *p* < 0.0001). A similar trend was observed for temporal closeness centrality (Figure 7B, *ρ* = 0.62, *p* < 0.0001). This entails that nodes appear to have similar centrality properties in the EO and EC resting-state conditions. Although both centrality measures showed between-session correlations, there was no consistent relationship between the two measures. No significant relation was observed in the EO session (Figure 7C, *ρ* = 0.09, *p* = 0.15), and a negative correlation emerged for the EC session (Figure 7D; *ρ* = 0.45, *p* < 0.0001). This result is not surprising, since the measures are quite different by definition, but it is still useful to demonstrate that these different centrality measures quantify different aspects of the temporal dynamics of the brain.

### Applying Burstiness

**Figure 8.**
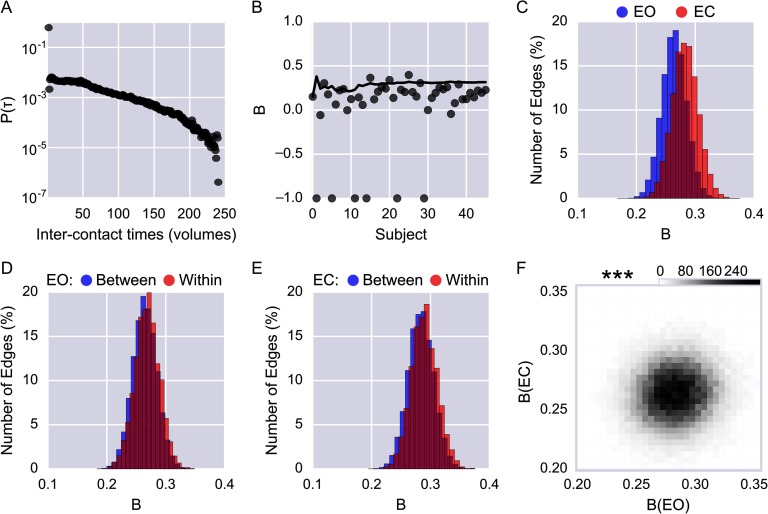
Quantifying bursty connectivity. (A) Distribution of all intercontact probabilities, combining all edges and subjects in the eyes-open (EO) condition. (B) The bursty coefficient (*B*) for one edge in the EO condition. Each dot represents *B* calculated per subject, while the solid line shows the bursty coefficient when cumulatively adding subjects. Values of − 1 indicate that all intercontact times are identical (i.e., one burst, tonic connectivity, or oscillations in connectivity). (C) Distributions of *B* for the different conditions (blue: EO; red: eyes-closed, EC). (D) Distributions of *B* as a function of EO within-network connectivity (red) and between-network connectivity (blue). (E) As in panel D, but for the EC condition. (F) Bursty coefficients for each edge across the two sessions, displayed as a heat map. *** signifies *p* < 0.001.

By applying the burstiness measure (*B*) to an fMRI dataset, we can ask questions related to the temporal distribution of brain connectivity. To illustrate that there is indeed a bursty pattern of brain connectivity, we first plotted the distribution of all intercontact times taken from all subjects and edges for the EO session and observed a heavy-tailed distribution (Figure 8A).

We then considered the question of the most robust way to calculate *B*, given that our example fMRI dataset had a rather low temporal resolution and only spanned a limited time period. It was possible that not enough edges might be present in each subject to allow a stable estimate of *B* for a single subject. To test this concern, we evaluated whether there was a difference in *B* for a single subject versus the concatenated intercontact times of multiple subjects. This was done for a single edge that connected right posterior cingulate cortex and right medial prefrontal cortex in the EO session. As is shown in Figure 8B, there is a considerable variance in the individual subject estimates of burstiness. If we cumulatively add subjects, however, the estimate of burstiness stabilizes after approximately 12 subjects. This illustrates the importance of having enough data to calculate reliable *B* estimates. Henceforth, all *B* estimates are calculated by pooling intercontact times over subjects.

We then wished to contrast EO versus EC in terms of burstiness. Both conditions showed bursty distributions across all edges (see Figure 8C), and slightly more so for the EC than for the EO condition. Both within- and between-network connectivity showed bursty distributions of connectivity patterns in both conditions (Figures 8D and 8E).

Given that both EO and EC showed bursty correlations, we tested whether the values of *B* correlated between conditions (Figure 8F). We found a weak, but significant, correlation between conditions (*ρ* = 0.066, *p* < 0.0001). This weak between-condition correlation (accounting for less than one percent of the variance, and probably driven by the number of data points) suggests that much of the variance of burstiness may have been task-specific. However, more research on this topic will be needed.

### Applying Fluctuability

Using the fluctuability measure, researchers may ask questions regarding how many unique edges exist in a temporal network model of the dynamic functional brain connectome, indicating whether more resources (i.e., diversity of connections) are required during a given task.

**Figure 9.**
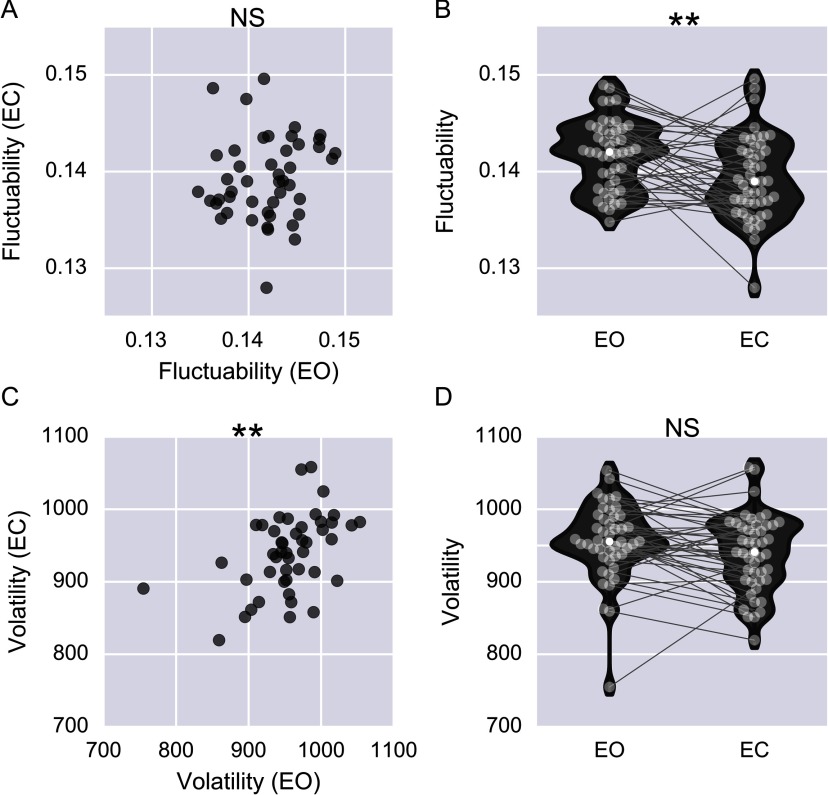
Applying fluctuability and volatility measures for the eyes-closed (EC) and eyes-open (EO) conditions. (A) Between-subjects correlation for fluctuability. (B) Violin plot showing fluctuability between the EO and EC conditions. Each light gray dot designates a subject, and a line binds together data obtained from the same subjects during the EO and EC conditions. For clarity, each line connecting subjects terminates at the centers of the violin plots. The mean value of fluctuability for each condition is shown with a white dot. (C) As in panel A, but for volatility. (D) As in panel B, but for volatility. ** signifies *p* < 0.01.

The fluctuability measure was applied to contrast the EO and EC conditions both between (Figure 9A) and within (Figure 9B) subjects. We observed no significant between-subjects correlation in *F* (*ρ* = 0.18 , *p* = 0.23) but did find a difference between the average values of *F* between conditions (p = 0.0020), with the EO condition having a higher degree of fluctuability. Thus, the EO condition had a more varying configuration of connections through time than did the EC condition.

### Applying Volatility

With volatility, we can ask whether the connectivity changes faster or slower through time. Some tasks might require the subject to switch between different cognitive faculties or brain states, while other tasks may require the brain to be more stable and to switch states less.

As with fluctuability, we computed volatility both between subjects (Figure 9C) and between conditions (Figure 9D). We observed a significant correlation for between-subject volatility over the two conditions (*ρ* = 0.46, *p* = 0.0012; Figure 9C). Additionally, no significant difference in volatility was observed between EO and EC (*p* = 0.051; Figure 9D).

### Applying Reachability Latency

The measure of reachability latency addresses the following question regarding the overall connectivity pattern along the temporal axis: how long does it take to travel to every single node in the temporal network? For example, the reachability latency may be useful for evaluating the dynamics when either functional or structural connectomes differ substantially. We computed the reachability latency by setting *r* = 1 (i.e., all nodes must be reached).

**Figure 10.**
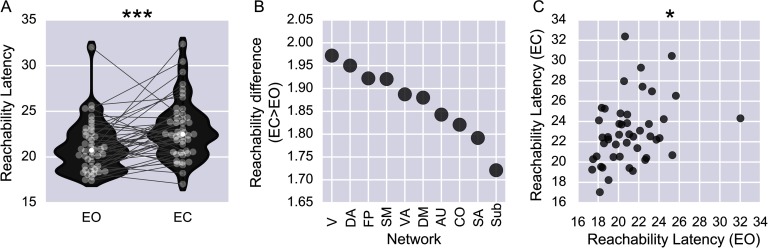
Applying reachability latency. (A) Violin plots of reachability latency for the eyes-open (EO) and eyes-closed (EC) conditions. Light gray dots correspond to single subjects, and lines connect each subject’s values between conditions. For clarity, each line connecting subjects terminates at the centers of the violin plots. White dots mark the mean reachability latencies. (B) Post hoc decomposition of the reachability latency difference (EC−EO) across subnetworks. (C) Between-subjects correlation of reachability between EO and EC. * signifies *p* < 0.05; *** signifies *p* < 0.001

The results are shown in Figure 10, where a significant difference in the average reachability latencies between conditions is visible (Figure 10A; EO: 21.07, EC: 22.96, *p* = 0.0005). Given that there was an overall increase in reachability latencies during EC as compared to EO, we decided to unpack this finding post hoc and check whether the discovered global difference in reachability could be localized to brain networks that should differ between the EC and EO conditions. So, rather than calculating the reachability latency for the entire brain, we averaged the measures of reachability latency (to reach all nodes) for ten preassigned brain networks. In this post hoc analysis, we see that the brain networks with the highest differences in reachability latency were the visual, dorsal attention, and frontoparietal brain networks (Figure 9B). Thus, the results show a longer reachability latency for the visual and attention networks when there is no visual input, a result that appears biologically plausible.

In addition to these between-condition differences in reachability, we observed that there was also a significant between-subjects relationship (*ρ* = 0.36, *p* = 0.015; Figure 9C). Taken together with the previous finding, our results show that measures of reachability latency reflect both between-task and between-subjects differences.

### Applying Temporal Efficiency

Finally, we computed the global temporal efficiencies for both the EO and EC conditions. Where reachability latency employs the shortest temporal path to calculate how long it takes to reach a certain percentage of nodes, temporal efficiency relates to the average inverse of all shortest temporal paths.

**Figure 11.**
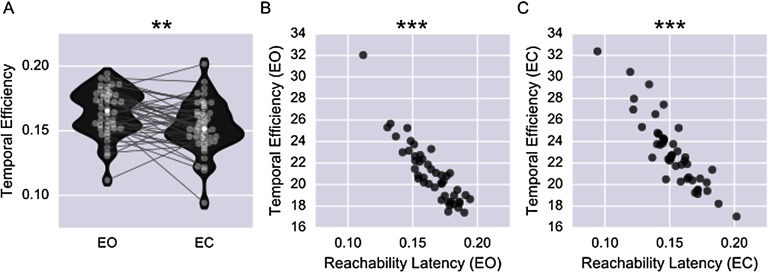
Applying global temporal efficiency and its relation with reachability latency. (A) Violin plots of global temporal efficiency for the eyes-open (EO) and eyes-closed (EC) conditions. Light gray dots correspond to single subjects, and lines connect single subjects, values between conditions. For clarity, each line connecting subjects terminates at the centers of the violin plots. White dots indicate the mean global temporal efficiencies. (B) Scatterplot showing each subject’s reachability latency versus temporal efficiency for the EO condition. (C) As in panel B, but for the EC condition. ** signifies *p* < 0.01; *** signifies *p* < 0.001.

We found that temporal efficiency was significantly greater during EO than during EC (*p* = 0.0011; Figure 11A). This finding means that, on average, the temporal paths are shorter in the EO than in to the EC condition. We observed strong negative correlations between temporal efficiency and reachability latency during both conditions (EO: *ρ* = −0.88, *p* < 0.0001; EC: *ρ* = −0.88, *p* < 0.0001; see Figures 11B and 11C).

## Discussion

Our overarching aim in this work was to provide an overview of the key concepts of temporal networks, for which we have introduced and defined temporal network measures that can be used in studies of dynamic functional brain connectivity. Additionally, we have shown the applicability of temporal metrics in network neuroscience by applying them fMRI resting-state datasets, and then shown that resting-state networks differ in their dynamical properties.

### Summary of Applying Temporal Network Measures to fMRI Data

Both temporal degree centrality and closeness centrality were correlated across conditions, whereas no correlation between the two centrality measures was observed. This entails that the two centrality measures quantify different dynamic properties of the brain.

At a global network level, we examined the temporal uniqueness of edges (fluctuability) as well as the rate of change of connectivity (volatility). We could identify a significant condition-dependent difference in fluctuability, but no difference was observed in volatility between conditions. Conversely, a significant between-subjects correlation was found for temporal network volatility, but the between-subjects correlation in fluctuability was not significant. The observed differences in volatility—that is, the differences in brain connectivity at different points in time—were driven to a relatively larger extent by intersubject differences in connectivity dynamics than by differences related to the tasks (EO/EC) per se.

Our results regarding reachability latencies during the EO and EC conditions indicate task-driven changes in latencies, especially since the connectivity of the visual and attention brain networks is known to reconfigure between EO and EC conditions (Zhang et al., 2015). Thus, the observed difference in reachability latencies might be a reflection of a putative network reconfiguration. Furthermore, reachability also showed a between-subjects correlation across conditions.

The distribution of intercontact time points of connectivity between brain nodes was bursty, in agreement with our previous findings (Thompson & Fransson, 2016a). Notably, our previous findings were obtained at a high temporal resolution (TR = 0.72 s), and it is therefore reassuring that we were able to detect similar properties of burstiness in brain connectivity at a lower temporal resolution (TR = 2 s). Of note, the between-network versus within-network connectivity difference here varied from that obtained in a previous study that had shown between-network connectivity to be significantly more bursty than within-network connectivity (Thompson & Fransson, 2016a). This difference is probably due to the different kinds of thresholding being applied. Here a variance-based thresholding was applied, instead of the magnitude-based form used in the previous study. We have discussed previously that these different strategies will prioritize different edges (Thompson & Fransson, 2015b, 2016b).

In sum, we have shown that measures founded in temporal network theory can be applied to fMRI data and are sensitive to the dynamical properties of fMRI BOLD connectivity. While attempting to interpret temporal network measures in a psychological and biological context has an intuitive appeal, such interpretations remain speculative at this point. Our intention here was to explore the dynamic connectivity across subjects and across a simple task difference to demonstrate that temporal network measures are appropriate measures given the signal. We showned that certain properties may be subject-specific, while others are task-specific. Furthermore, temporal network measures lead to rankings of network properties that are in agreement in a resting state during eyes-open and eyes-closed conditions. However, to infer psychological properties from a specific measure, hypothesis-driven work will be necessary in which the a priori hypothesis can be explicitly tested. We believe that the present work demonstrates that such future studies are possible using temporal network theory.

A final note regarding the interpretation of measures that are dependent on the shortest paths. In cases in which the temporal resolution is sluggish, as in fMRI, and the spatial resolution is coarse in relation to the size of neurons, it would be incorrect to assume that the shortest path reflects the shortest period of time for a neuron in node *i* to reach and communicate with another neuron residing in node *j*. What can the shortest paths reveal, then? When the temporal paths decrease during performance of a given task between nodes located in two different brain networks, it tells us that those two networks are interacting. Thus, shortest paths computed on the basis of fMRI data should be viewed in the context of how nodes in different brain networks are interacting with each other.

### Other Approaches to Remporal Network Theory

The list of measures for temporal networks described here is far from exhaustive. Although we have focused primarily on temporal properties that can be defined at a nodal and/or global level, detecting changes in network modularity over time is an active part of network theory research (Mucha, Richardson, Macon, Porter, & Onnela, 2010; Rosvall & Bergstrom, 2010). This approach has recently been applied to the brain connectome (Mantzaris et al., 2013; Bassett et al., 2013) and in the context of learning (Bassett et al., 2011; Basset, Yang, Wymbs, & Grafton, 2015). In a similar vein, the presence of hyperedges allows us to explore and identify groups of edges that have similar temporal evolutions (Davison et al., 2015; Davison et al., 2016). Similarly, investigating how different tasks evoke different network configurations (Ekman, Derrfuss, Tittgemeyer, & Fiebach, 2012; Cole et al., 2013; Matter, Cole, & Sharon, 2015) is also an active research area. Another recent exciting development is to consider a control-theory-based approach to network neuroscience (Gu et al., 2015), which can be applied to networks embedded in time (Gu et al., 2016).

Yet another avenue of temporal network research is to apply static network measures to each t-graphlet and then derive time series of fluctuating static measures (Bola & Sabel, 2015; Chiang et al., 2016). It is also possible is to quantify the properties of dynamic fluctuations in brain connectivity through time and then to correlate them with the underlying static network. Using such a strategy, between-subjects differences for both the dynamic and the static networks can be revealed (e.g., Betzel, Fukushima, He, Zuo, & Sporns, 2016).

Finally, considerably more measures within the temporal network literature can be put to use within the field of network neuroscience. For example, the list of centrality measures provided here is not complete. A temporal extension of betweenness centrality, which is often used for static networks, can be adopted in the temporal domain (Tang, Musolesi, Mascolo, Latora, & Nicosai, 2010). In the same vein, spectral centrality can also be computed in the temporal domain (see Nicosia et al., 2013, for further details).

### When Is Temporal Network Theory Useful?

As we stated in the introduction, graphs are an abstract representation corresponding to some state in the world. The properties quantified in these representations try to reflect corresponding properties of the world. Not every representation of brain function will require time, which would make temporal network measures unsuitable. Under what conditions will temporal network theory be of use? Networks of neurons are known to reconfigure during different behavioral states and tasks. This reconfiguration occurs at all levels of brain connectivity: from microcircuits controlling the digestive system in lobsters (Meyrand, Simmers, & Moulins, 1994) to the differential involvement of large-scale brain networks in cognitive tasks in humans (Ekman et al., 2012; Cole et al., 2013; Mattar, Cole, & Sharon, 2014). Temporal network theory allows us to track and study these reconfigurations, which has the potential to offer more detailed information about fluctuations in brain network configuration than can be achieved by aggregating over tasks or behavioral states.

The potential fields of application for the methods described in this work are vast. Obvious examples range from real-time neuroimaging to tracking ongoing cognitive processes. To be able to isolate and identify the dynamics of networks may indeed be necessary when the same networks are involved in multiple cognitive processes. Furthermore, the increased temporal sensitivity provided by temporal networks offers greater systematic discriminative power between healthy and patient cohorts. To give an example, alterations in static default mode network connectivity have been implicated in depression (Sheline et al., 2009; Hamilton et al., 2012), schizophrenia (Garrity et al., 2007; Pomarol-Clotet et al., 2008), traumatic brain injury (Bonnelle et al., 2011; Sharp et al., 2011; Thompson, Thelin, Lilja, Bellander, & Fransson, 2016), obsessive–compulsive disorder (Stern, Fitzgerald, Welsh, Abelson, & Taylor, 2012), autism (Cherkassky, Kana, Keller, & Just, 2006; Weng et al., 2010), fibromyalgia (Nadapow et al, 2010; Flodin et al., 2014), posttraumatic stress (Sripada et al., 2012), and Alzheimer’s disease (Greicius, Srivastava, Reiss, & Menon, 2004). This is not an exhaustive list, but it list suggests that it is very difficult to make inferences regarding static differences in connectivity in the default network that are specific to a particular patient cohort. We hope that, by adding a temporal dimension and thus reducing the aggregations and simplifications of a static network analysis, unique connectivity markers may become viable for different patient cohorts.

A couple of additional Factors should be considered when applying temporal network theory. Interpreting what a measure means can only be done in relation to the temporal resolution of the data. For example, volatility will obviously entail a different interpretation when it is applied to a dataset obtained with a temporal resolution of years versus a dataset acquired with a temporal resolution of milliseconds. Furthermore, measures using shortest temporal paths can be altogether inappropriate in certain situations (e.g., longtitudinal studies).

Finally, consideration is also needed about which temporal network measure(s) should be applied to a research question. Although temporal network theory puts a wide array of measures at the user’s disposal, we advise against applying the entire battery of measures to a given dataset. Given a hypothesis about some state of the world (*S*), this should first be translated into a hypothesis about which network measure will quantify the network representation of *S*. A more exploratory analysis showing significant (and multiple-comparison-corrected) correlations in five out of ten measures, when these measures were not first formulated in relation to *S*, may become hard, if not impossible, to translate into something meaningful.

### Limitations and Extensions for Temporal Network Measures

Our scope was limited to temporal measures that operate on binary time series of brain connectivity (i.e., binary t-graphlets). Most of the measures discussed here can be extended and defined for series of weighted connectivity matrices. However, certain temporal measures are not straightforward to convert to the weighted case. Pertinent examples are burstiness and reachability for which no simple strategy has been identified to apply them in a weighted-connectivity context.

Regardless of the method used to derive the brain connectivity time series, it is important that adequate preprocessing steps be performed on the data to avoid potential bias in the analysis. Our method of deriving t-graphlets with weighted Pearson correlation coefficients to compute time series of brain connectivity is not exempted from this concern. In a connectivity analysis based on sequences of binary t-graphlets, the absence or presence of an edge might potentially be influenced by the user’s selection of thresholding. Hence, the strategy regarding how to optimally threshold the t-graphles into binary graphlets is of vital importance. We believe that it is important to keep in mind that comparisons of the variances as well as the means of connectivity time series might be biased by the underlying mean–variance relationship (Thompson & Fransson, 2015b, 2016b). This further emphasizes the need for adequate thresholding strategies for connectivity time series. Moreover, subject head motion, known to be a large problem for fMRI connectivity studies (Dijk et al., 2012; Power et al., 2012, Power, Schlaggar, & Petersen, 2015), can also lead to spurious dynamic properties (Laumann et al., 2016).

By providing a survey of the theory of temporal networks and showing their applicability and usefulness in network neuroscience, we hope that we have stirred the reader’s interest in using models based on temporal networks when studying the dynamics of functional brain connectivity. To this end, we have implemented all temporal network measures described in the present article in a software package that is freely available (Teneto, which is written in Python and can be downloaded at http://github.com/wiheto/teneto). We plan to extend the Teneto package to include additional temporal network measures, plotting routines, wrappers for other programming languages, and dynamic connectivity estimation.

## ACKNOWLEDGMENTS

We thank Pontus Plavén-Sigray, Bjärn Schiffler, Granville Matheson, Simon Skau, and Lieke de Boer for helpful comments and discussions about the manuscript. This work was supported by the Swedish Research Council (Grant Nos. 621-2012-4911 and 013-61X-08276-26-4) and the Swedish e-Science Research Center.

## AUTHOR CONTRIBUTIONS

William Hedley Thompson: Conceptualization; Formal analysis; Investigation; Methodology; Software; Visualization; Writing – original draft; Writing – review & editing Per Brantefors: Conceptualization; Methodology; Writing – review & editing Peter Fransson: Conceptualization; Funding acquisition; Investigation; Methodology; Supervision; Writing – original draft; Writing review & editing.

## TECHNICAL TERMS

Temporal network: A multigraph whose edges contain temporal information
Multigraph: A network whose nodes can be connected by multiple edges
Graphlet: A discrete connectivity matrix that expresses part of a larger network
Shortest temporal path: The shortest path by which a node communicates with another node across multiple intermediate nodes
Intercontact time: The time taken between two nodes with a direct connection
Weighted Pearson correlation: A correlation coefficient for which the significance of each observation is determined by a weight
